# Molecular adaptation to salinity fluctuation in tropical intertidal environments of a mangrove tree *Sonneratia alba*

**DOI:** 10.1186/s12870-020-02395-3

**Published:** 2020-04-22

**Authors:** Xiao Feng, Shaohua Xu, Jianfang Li, Yuchen Yang, Qipian Chen, Haomin Lyu, Cairong Zhong, Ziwen He, Suhua Shi

**Affiliations:** 1 State Key Laboratory of Biocontrol, Guangdong Key Lab of Plant Resources, Key Laboratory of Biodiversity Dynamics and Conservation of Guangdong Higher Education Institutes, School of Life Sciences, Sun Yat-Sen University, Guangzhou, China; 2 Hainan Dongzhai Harbor National Nature Reserve Administration, Haikou, China

**Keywords:** Homeostasis, Mangrove, Salt adaptation, *Sonneratia alba*, Transcription factor, Transcriptome profiles

## Abstract

**Background:**

Mangroves have adapted to intertidal zones - the interface between terrestrial and marine ecosystems. Various studies have shown adaptive evolution in mangroves at physiological, ecological, and genomic levels. However, these studies paid little attention to gene regulation of salt adaptation by transcriptome profiles.

**Results:**

We sequenced the transcriptomes of *Sonneratia alba* under low (fresh water), medium (half the seawater salinity), and high salt (seawater salinity) conditions and investigated the underlying transcriptional regulation of salt adaptation. In leaf tissue, 64% potential salinity-related genes were not differentially expressed when salinity increased from freshwater to medium levels, but became up- or down-regulated when salt concentrations further increased to levels found in sea water, indicating that these genes are well adapted to the medium saline condition. We inferred that both maintenance and regulation of cellular environmental homeostasis are important adaptive processes in *S. alba*. i) The sulfur metabolism as well as flavone and flavonol biosynthesis KEGG pathways were significantly enriched among up-regulated genes in leaves. They are both involved in scavenging ROS or synthesis and accumulation of osmosis-related metabolites in plants. ii) There was a significantly increased percentage of transcription factor-encoding genes among up-regulated transcripts. High expressions of salt tolerance-related TF families were found under high salt conditions. iii) Some genes up-regulated in response to salt treatment showed signs of adaptive evolution at the amino acid level and might contribute to adaptation to fluctuating intertidal environments.

**Conclusions:**

This study first elucidates the mechanism of high-salt adaptation in mangroves at the whole-transcriptome level by salt gradient experimental treatments. It reveals that several candidate genes (including salt-related genes, TF-encoding genes, and PSGs) and major pathways are involved in adaptation to high-salt environments. Our study also provides a valuable resource for future investigation of adaptive evolution in extreme environments.

## Background

How species adapt to extreme environments is a fundamental topic of evolutionary biology. Located at the interface between terrestrial and marine ecosystems, intertidal zones impose many harsh or extreme conditions on local organisms, including high salinity, hypoxia, salt fluctuations, strong ultraviolet (UV) light, and high temperature [1]. Nevertheless, mangroves have colonized and became well adapted to these habitats, evolving a series of highly specialized traits including salt tolerance, viviparous embryos, aerial roots, and high tannin content [2–4]. Mangroves are a major group of marine halophytes that thrive and complete their life cycles in high-salinity environments that arise due to fluctuating seawater levels. Thus, they are good model systems for investigating adaptive evolution. Indeed, adaptation in this group has been studied at the phenotypic, ecological, physiological, and genomic levels [5–10]. However, little attention has been paid to transcriptional regulation even though it has been strongly selected for rapid adaptation to changes in environmental conditions and may play a critical role at ecological and evolutionary timescales [11].

Salt adaptation is a long-term and dynamic process brought about by multiple genes involved in many morphological, physiological, molecular, and cellular processes [12–14]. Various salt-responsive genes and important signaling pathways have been identified by global gene profiling in the model plant *Arabidopsis thaliana* [12, 15], and crop plants rice [16] and soybean [17]. The Salt overly sensitive (SOS) signaling pathway, Ca^2+^-dependent signaling pathways, and ABA signaling pathways contribute to mediating cellular signaling under salt stress and maintain cellular environmental homeostasis [18]. Many molecular mechanisms have been revealed in glycophytes, but the major limiting factor is their inability to survive high and fluctuating salinity. These studies are insufficient to understand molecular aspects of plant salt adaptation.

*Sonneratia alba* inhabits low intertidal zones of downstream estuarine systems and is one of the most pervasive and salt tolerant mangrove species. It reaches an optimal growth in 5 to 50% seawater, indicating its capacity to tolerate high salinity and hypoxia [19]. It can synthesize organic solutes to adjust cellular osmotic potential and produce antioxidant enzymes for scavenging reactive oxygen species (ROS) against high salinity [20]. Therefore, it is an attractive ecological model to investigate genetic mechanisms underlying salt adaptation. However, only limited RNA-seq data from *Sonneratia* have been reported to date [21, 22]. The lack of transcriptomic information for *S. alba* across salinity conditions is a major obstacle to discerning the molecular bases of adaptation to saline environments. In this study, we designed an experiment to elucidate the transcriptional regulation of high-salt adaptation in *S. alba*. We sequenced transcriptomes from leaves and roots of *S. alba* seedlings irrigated with artificial seawater. After genome-guided transcriptome assembly, we characterized the transcriptomes under low, medium, and high salinity conditions for further functional analyses. We wish to gain new insights into the potential genetic, biochemical, and physiological mechanisms of salt adaptation in *S. alba*. Our study also generates valuable resources to support further research into adaptive evolution of mangroves in extreme environments.

## Results

### Identification of differentially expressed genes and expression patterns

To generate genomic resources and begin elucidating the mechanisms of salt tolerance in *S. alba*, we performed RNA-seq experiments on leaves and roots from plants reared in media with various salt levels. The read summary statistics and assembly results are outlined in Table 1. We generated 44.20 to 61.97 million of 100 bp paired-end reads from 12 libraries. After quality filtering (see Methods for details) we retained 41.94 to 59.21 million 99 bp high quality reads and successfully mapped 93.85 to 94.83% of them to the *S. alba* reference genome. These aligned reads were assembled separately within each sample, yielding 33,930 to 40,937 transcripts with average lengths 3298 to 3530 bp and N50 values of 4214 to 4437 bp (Table 1). The correlation coefficients between biological replicates in each condition were from 0.87 to 0.97 (Additional file 1: Figure S1).

**Table 1:** Summary of mapping and assembly statistics of the *Sonneratia alba* transcriptomes

Sample	Replicate	Mapping to genome		Multiple mapped		Uniquely mapped		Number of transcripts	Average size (bp)	N50 (bp)
		Number (× 10^6^)	%	Number (× 10^6^)	%	Number (× 10^6^)	%			
L0	r1	52.26	94.59	0.44	0.84	51.82	99.16	36,664	3395	4301
	r2	42.27	94.12	0.64	1.51	41.64	98.49	35,970	3378	4235
L250	r1	39.85	94.02	0.42	1.05	39.43	98.95	33,930	3318	4214
	r2	39.36	93.85	0.61	1.54	38.75	98.46	35,402	3394	4273
L500	r1	55.66	94.01	0.59	1.06	55.08	98.94	34,116	3298	4221
	r2	43.41	93.99	0.87	2.01	42.54	97.99	34,334	3443	4338
R0	r1	45.47	93.97	0.49	1.09	44.97	98.91	40,937	3471	4352
	r2	43.01	94.43	1.92	4.47	41.08	95.53	40,005	3469	4380
R250	r1	44.20	94.48	0.49	1.12	43.71	98.88	40,696	3425	4363
	r2	40.78	94.58	1.99	4.88	38.79	95.12	40,483	3476	4383
R500	r1	52.23	94.58	0.77	1.47	51.46	98.53	40,338	3530	4437
	r2	40.63	94.83	1.13	2.79	39.49	97.21	39,426	3503	4391

L and R represent leaf and root tissues; 0, 250 and 500 represent the salinity treatments (mM NaCl); r1 and r2 indicate two biological replicates with three individuals each. For each sample, RNA-seq reads mapping and genome-guided transcriptome assembly were performed individually

**Fig. 1:**
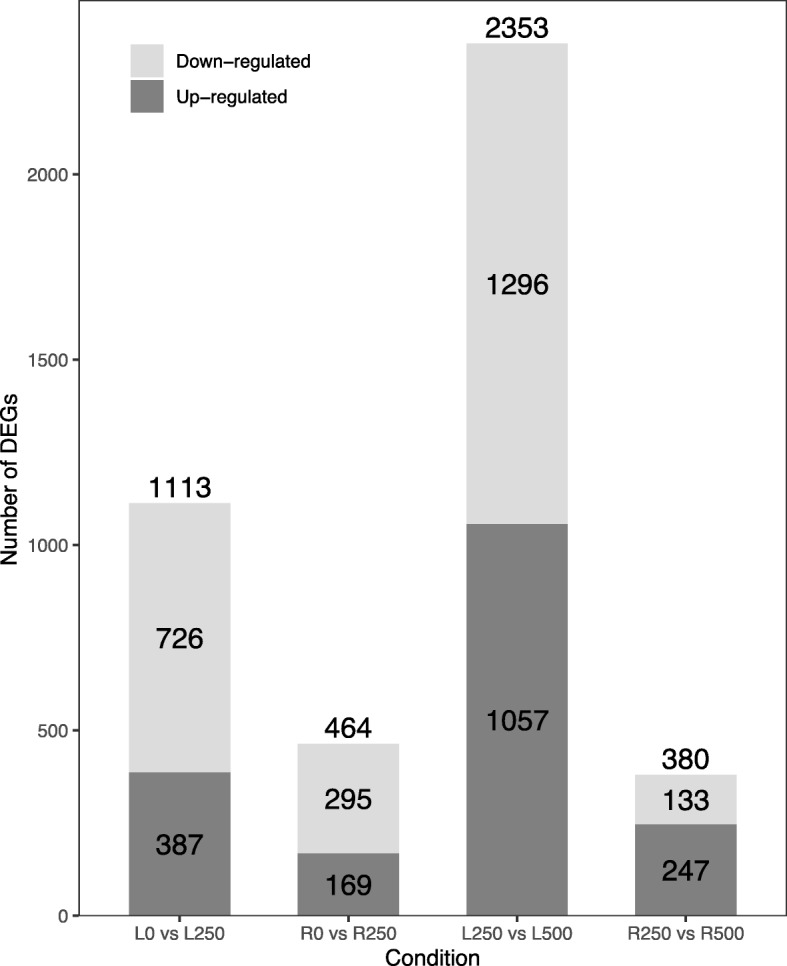
Differentially expressed genes (DEGs). The number of up- and down-regulated genes in leaves and roots across salinity conditions (0 mM vs 250 mM NaCl and 250 mM vs 500 mM NaCl) is shown. The total number of DEGs is listed on top of each bar

For *S. alba*, its adaptation to the intertidal zone environment is a long-term and dynamic process, and its growth environment is 5 to 50% seawater concentration, that is, 25 mM to 250 mM [19]. Hence, 500 mM and 0 mM provide the hyper-saline (stressful) and low-saline conditions, respectively. A large number of genes between 0 vs 250 are expected to have similar expression levels. The stress would be between 250 mM and 500 mM. We next performed two pair-wise comparisons of transcript abundance: low (0 mM) vs medium (250 mM), and medium vs high (500 mM) salt concentrations (see Methods for details). In the low to medium NaCl comparison, we found 387 significantly up-regulated and 726 down-regulated genes in leaves, and 169 up- and 295 down-regulated transcripts in roots. We found 1057 up- and 1296 down-regulated genes in leaves, and 247 up- and 133 down-regulated transcripts in roots when comparing the medium and high salt conditions (Fig. 1). There were clearly more differentially expressed genes (DEGs) in leaves than in roots. Moreover, we found more up-regulated genes in the medium to high salt contrast than in the low to medium comparison.

**Fig. 2:**
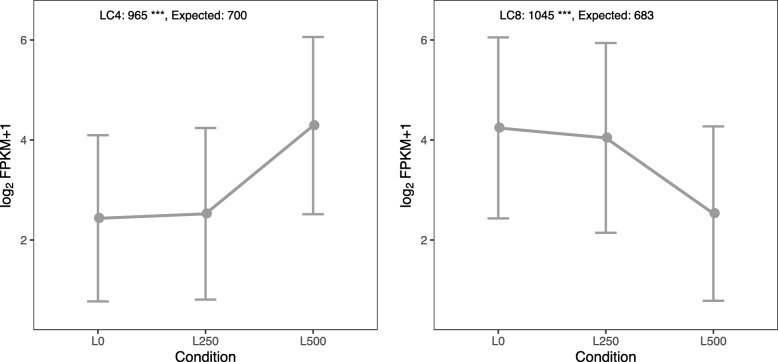
Significant expression clusters in leaves. The lines represent mean expression levels (means of log_2_(FPKM value + 1)) of all genes in a cluster. The error bars represent the standard deviation. The observed and expected numbers of genes belonging to each cluster are labeled on the top of each graph. A permutation test was used to evaluate the significance of differences. The clusters with statistically significant difference in the number of DEGs are marked with triple (*P*-value < 0.01) asterisks

To identify co-expression patterns among the DEGs, we clustered them into eight groups according to their expression change across salinity conditions and tissues (Additional file 2: Figure S2). Intriguingly, the two largest leaf clusters, LC8 (1045 genes) and LC4 (965 genes), comprised only genes that are significantly down- or up-regulated under high salt treatment (Fig. 2). The same pattern did not hold in the roots, however. To assess whether the LC8 and LC4 clusters are larger than expected by chance, we conducted a permutation test (see Methods for details). We indeed saw that these clusters were larger than expected (Fig. 2). The most genes didn’t differentially express across salinity conditions. The LC4 and LC8 are majority (64%) of all eight clusters. They are only differentially expressed when the salt levels become high (500 mM), but are unchanged in the medium to low NaCl comparison. Thus, only the extremely high levels of salt treatment were able to appreciably change the *S. alba* transcriptome, as expected given the high salinity tolerance of this species.

### Functional enrichment and transcriptional regulation

**Fig. 3:**
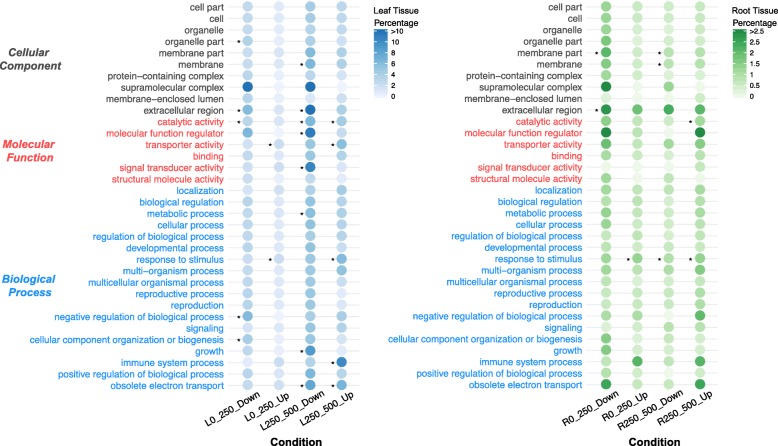
The GO representation analyses. All of GO terms at level 2 were shown. The bubble diagrams are GO terms overrepresented among up- and down-regulated genes in leaves (left) and roots (right) under different salt level contrasts. The darker the color bubbles, the higher the proportion of up- or down-regulated genes in the GO term. The significantly enriched GO terms are marked with asterisks (*P*-value < 0.05)

We assigned functional Gene Ontology (GO) categories to the DEGs we identified (Additional file 3: Table S1) and four major co-expression clusters, including LC4, LC8, LC4-RC4 (overlaps between LC4 and RC4), LC8-RC8 (Additional file 4: Table S2). Using a whole-genome annotation as the background, we found several categories that were over-represented among our differentially expressed genes (Fig. 3). Remarkably, many of the genes up-regulated in leaves were involved in the molecular function category “transporter activity.” The biological process category “response to stimulus” was significantly overrepresented in all four comparisons (both leaves and roots). Furthermore, many up-regulated genes were assigned to the “response to stimulus” category in four comparisons (57 in L0 vs L250, 35 in R0 vs R250, 158 in L250 vs L500, 36 in R250 vs R500). Detailed annotations were also performed across up-regulated genes. We note that many genes were annotated with functions involving salt tolerance, especially Sodium/hydrogen exchanger (NHX) encoding gene (*SA_23236* in L0 vs L250 and L250 vs L500, *SA_23120* in R250 vs R500), Tonoplast intrinsic protein (TIP) encoding gene (*SA_14179* in L250 vs L500), Plasma membrane intrinsic protein (PIP) encoding gene (*SA_2543*1 in L0 vs L250 and L250 vs L500, *SA_09545* in L0 vs L250, *SA_08276*, *SA_20957*, *SA_24093*, *SA_26218*, *SA_26346* in L250 vs L500), *Mangrin* (*SA_08086*, *SA_14631* in L250 vs L500), several heat shock protein encoding genes, and main transcription factor encoding genes (described in the following section). *Mangrin* is partially homologous to the allene oxide cyclase (AOC) encoding gene. Several genes up-regulated under high salt directly adjust cellular osmotic potential or integrate metabolic, hormonal, and environmental signals in stress acclimatization. They include the glutathione peroxidase encoding gene (*SA_08946* in roots and *SA_18901* in leaves), the monodehydroascorbate reductase encoding gene (*SA_20647* in leaves), the aldo-keto reductase encoding gene (*SA_02762* in both tissues, *SA_02758*, *SA_02759* in leaves, and *SA_15935* in roots), a number of key enzymes in biosynthesis of asparagine, cysteine, galactinol, trehalose-6-phosphate, and stachyose. We also found up-regulated transcripts of unknown function. These can be further investigated to gain fresh insights in into salt adaptation.

Interestingly, 965 of the transcripts up-regulated in leaves fall into the LC4 cluster, as do 89.24% of the 158 genes that were both up-regulated and assigned to the “response to stimulus” GO category (Additional file 4: Table S2). This suggests that this cluster plays an important role in response to abnormally high salt stress.

The GO terms significantly enriched among down-regulated genes were “extracellular region” and “catalytic activity” categories in leaves and “membrane part” in roots (Fig. 3). Furthermore, 1045 of transcripts down-regulated in leaves fall into the LC8 cluster, were enriched in categories of “carbohydrate metabolic process”, “cell periphery”, “negative regulation of molecular function”, “catalytic activity” and so on (Additional file 4: Table S2). They were closely associated with basic metabolism, plant growth regulation, molecular function regulator, signal transducer activity, and electron transport.

We next investigated metabolic pathways significantly enriched among the DEGs by cross-referencing our list of up-regulated genes with the KEGG annotation of the *S. alba* reference genome (Additional file 5: Table S3). Of the metabolic pathways overrepresented among transcripts differentially expressed under high salt (four highly and three slightly enriched, Table 2), sulfur metabolism, protein processing in endoplasmic reticulum, and flavone and flavonol biosynthesis were the most interesting because they are known to be involved in *S. alba* salt adaptation. Flavonoid biosynthesis, nicotinate, and nicotinamide metabolism pathways were also overrepresented among genes up-regulated under high salt treatment in roots (Table 2). No metabolic pathways stood out among genes differentially expressed under the medium compared to low salt conditions.

**Table 2:** KEGG pathways significantly enriched among up-regulated genes in leaves and roots (250 mM vs 500 mM)

Condition	Pathway ID	Pathway	No. of genes in genome	No. of up-regulated genes	*P* value	Adjusted *P* value
Leaf	ko00920	Sulfur metabolism	121	19	7.12E-08	1.58E-05
	ko04141	Protein processing in endoplasmic reticulum	605	46	2.41E-06	1.79E-04
	ko04626	Plant-pathogen interaction	1253	74	3.61E-05	2.01E-03
	ko00944	Flavone and flavonol biosynthesis	101	13	7.10E-05	3.15E-03
	ko00592	alpha-Linolenic acid metabolism	95	10	2.28E-03	6.32E-02
	ko00760	Nicotinate and nicotinamide metabolism	53	7	2.84E-03	7.01E-02
	ko00270	Cysteine and methionine metabolism	312	22	3.13E-03	6.96E-02
Root	ko00941	Flavonoid biosynthesis	85	5	7.78E-04	9.80E-02
	ko00760	Nicotinate and nicotinamide metabolism	53	4	1.05E-03	6.60E-02

**Fig. 4:**
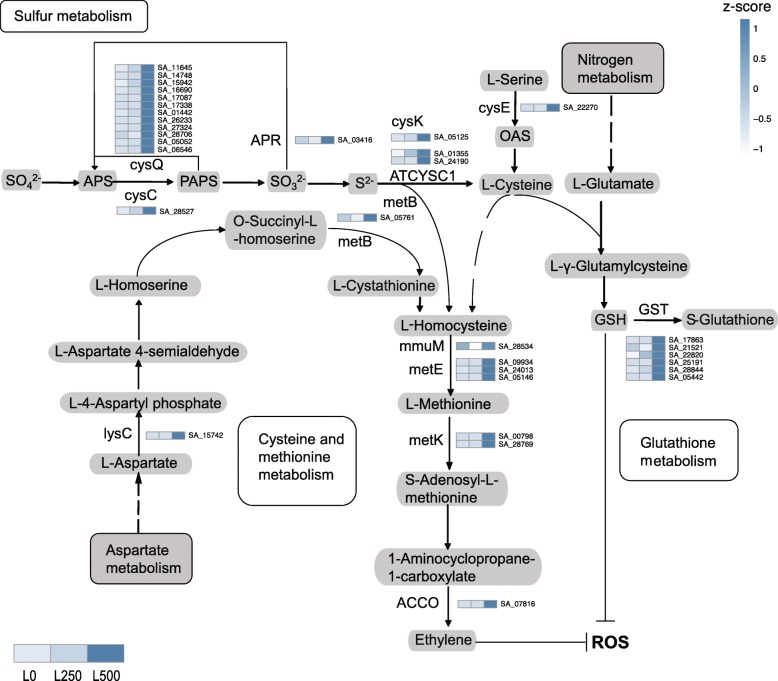
Major metabolic pathway regulation under different salt levels in leaves. Expression profiles of up-regulated genes involved in sulfur, glutathione, cysteine, and methionine metabolic pathways are shown in heatmaps. The scale on the top right represents FPKM values with z-score normalization

To further explore the regulation of biological pathways under salt treatment, we visualized the expression profiles of up-regulated genes in transcripts assigned to major pathways (Fig. 4). The highlighted pathways are involved in reactive oxygen species (ROS) scavenging, detoxification, and regulation of cell osmotic pressure. We reason that these pathways play essential roles in salt adaptation. Notably, these functional enrichment analyses of DEGs point to the cellular environment as the possible site of molecular salt adaptation in *S. alba*. This seems plausible as changes in candidate proteins occur within cells.

### Transcription factors involved in salt adaptation

**Fig. 5:**
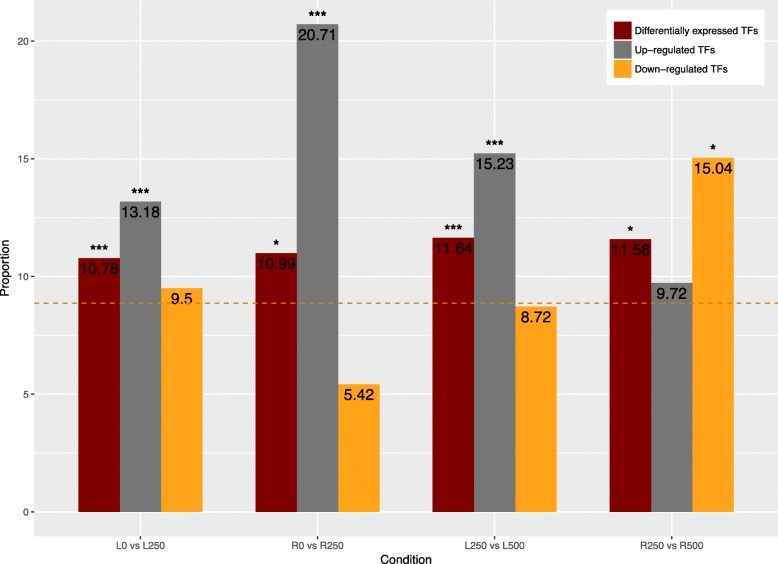
The proportions of differentially expressed transcription factor encoding genes in leaves and roots under different salinity contrasts (0 mM vs 250 mM NaCl and 250 mM vs 500 mM NaCl). Orange dashed line represents the proportion of transcription factor encoding genes in the whole *S. alba* genome. Red bars represent the proportion of differentially expressed TFs among all DEGs. Gray bars represent the proportion of TFs among up-regulated genes. Orange bars represent the proportion of TFs among down-regulated genes. Fisher’s exact test was used to evaluate the significance of the differences between the proportions of differential expressed / up-regulated / down-regulated transcription factors and transcription factor encoding genes in the whole genome. *P*-value ranges are denoted with single (corrected *P*-value < 0.1) or triple (corrected *P*-value < 0.05) asterisks

Transcription factors (TFs) are regulatory proteins and therefore have the potential to regulate multiple aspects of salt adaptation by influencing several downstream factors. Previous studies have revealed that TFs perform important functions in abiotic stress response and adaptation [23–27]. Intriguingly, we found more transcription factors, as a proportion of all annotated genes, in *S. alba* (2595, 8.86%) and *S. caseolaris* (2729, 9.72%) than in their inland relatives *P. granatum* (1847, 6.34%) and *E. grandis* (2185, 6.01%). Further, TF encoding genes were overrepresented among the differentially expressed genes identified in all contrasts we performed, but particularly in leaves. The pattern of up-regulated TFs was significant in three comparisons: both leaf contrasts and the low vs medium NaCl in roots, and several down-regulated TFs slightly enriched in the comparison between medium and hyper saline conditions in roots (Fig. 5). Separating transcription factors into generally recognized families (Additional file 6: Figure S3), we found that the *AP2*/*EREBP*, *NAC*, *WRKY*, and *bZIP* families were enriched among the up-regulated genes.

### Positive selection in up-regulated genes

Unusual salt tolerance is a derived characteristic of mangroves. The Ka/Ks values along *S. alba* and its relatives were calculated (Additional file 7: Figure S4). It showed that Ka/Ks ratio was elevated in the *S. alba* relative to relatives. We would therefore expect that some of the genes up-regulated in response to NaCl treatment have acquired their roles recently in evolutionary time. We wondered if their amino acid composition changed along with expression level. To test this idea, we used the PAML package to identify positively selected genes (PSGs) in *S. alba* (see Methods for details). We identified sixteen up-regulated PSGs (Additional file 8: Table S4). The *SA_12151* locus has particularly high Ka/Ks ratio (Ka/Ks = 4.42) and has probably undergone positive selection. Functional annotation gives us further confidence that these proteins are relevant to salinity resistance.

### Real-time quantitative PCR (qPCR) DEG verification

**Fig. 6:**
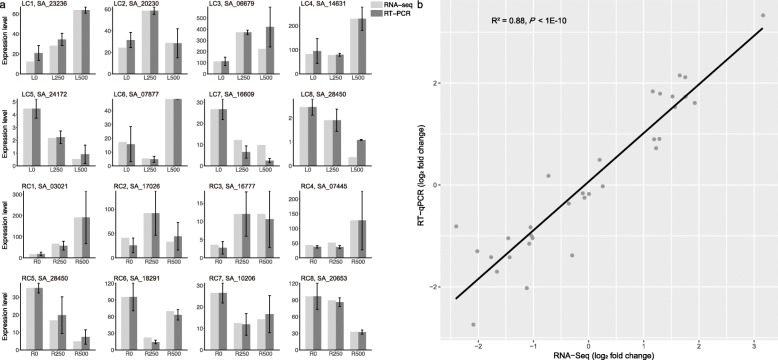
Validation of RNA-seq results by RT-qPCR. **a** Transcript levels of 16 genes in the three samples were detected by RT-PCR. Light grey bars indicate transcript abundance changes estimated from RNA-seq. Dark grey bars with associated standard deviations represent the normalized relative expression levels determined by real-time quantitative PCR using the 2^-△△CT^ method. **b** Correlation of gene expression levels between RNA-seq and RT-qPCR data

To assess the reliability of RNA sequencing for DEG identification, transcripts of 16 genes from each cluster were evaluated by qPCR. The expression patterns of selected genes were consistent with the RNA-seq data in eight clusters from both leaves and roots. The results of qPCR are shown in Fig. 6a. The correlation between the log_2_ fold change of expression level from the RNA-seq experiments and qPCR is shown in Fig. 6b. Correlation between these methods is strong and positive (R^2^ = 0.88, *P*-value <1E-10), suggesting that RNA-seq results are highly reproducible.

## Discussion

Comparative genomics has been extensively used to investigate the genetic bases of adaptation to extreme environments [6, 7, 9, 10, 14, 28–31]. In this study, comprehensive transcriptome analyses from leaves and roots of *S. alba* across salinity conditions were performed to probe the genetic mechanisms underlying salt adaptation. Our results yielded candidate genes and mechanistic hypotheses that may deepen our understanding of salt adaptation in halophytes and provide a resource for further studies.

Salt adaptation is both a long-term and dynamic process influenced by multiple genes and involves many morphological, physiological, molecular, and cellular processes [12, 14, 32–34]. The requirement of bulk protein synthesis as part of the global defense strategy of *Arabidopsis* has been extensively shown in previous studies [35]. In *Zostera marina*, which experiences the full marine seawater, Na^+^ and K^+^ antiporters were displayed as a typical component. The salt-tolerant H^+^-ATPase plays an important role in maintaining the low Na^+^ concentration in the seagrass cells since it is strongly expressed in vegetative tissue [36, 37]. And nitrate assimilation and ion compartmentalization are also required in the process of adaptation [38]. While mangroves induce genes that function in specific salt-responsive pathways and transcription factors that influence the cellular environment. Most genes differentially expressed across salt levels in our study were induced or suppressed by the highest salt concentration in leaves. The pattern is different in roots, suggesting that salt adaptation in *S. alba* is tissue-specific.

A major group of up-regulated genes identified in our study were involved in ion transportation and osmotic regulation, including *NHX*, *TIP*, *PIP*, *Mangrin*. These genes have been reported to play major roles in salt tolerance in a variety of plants [39–42]. Particularly, *Mangrin* is partially homologous to the allene oxide cyclase (AOC) encoding gene. It is up-regulated by high salinity and its overexpression enhances salt tolerance in transgenic yeast and tobacco cells [39]. Most transcripts down-regulated under high salt conditions were involved in basic metabolism, plant growth regulation, molecular function regulator, signal transducer activity, and electron transport. This suggests that the long-term high salt treatment might suppress normal metabolic processes and throw off the plant homeostatic balance. Indeed, high salinity is an important agricultural contamination that result in damage to the plant [43]. In *Arabidopsis*, salt stress does not simply decrease the steady-state growth rates. While the growth of several discrete phases was strongly suppressed by salt stress, the others were actually increased [44].

Hypersaline stress always leads to secondary oxidation stress, resulting in reactive oxygen species accumulation. We indeed observed that a number of key enzymes (such as cysQ, ATCYSC1, cysK, GST) involved in sulfur, cysteine and methionine, and glutathione metabolism were up-regulated in leaves (Fig. 4). These pathways enhance synthesis of sulfur-rich peptides (such as S-Glutathione) to scavenge ROS [45]. In addition, we found that flavone and flavonol biosynthesis pathway genes were overrepresented in our set of differentially expressed transcripts. Flavonoids are natural antioxidants that can reduce ROS levels and aid in survival and growth of plants [46].

Recycling of nicotinamide and nicotinate for pyridine nucleotide synthesis can be stimulated by salt to protect pyrimidine and purine derivatives for nucleotide synthesis in the mangrove *Bruguiera sexangula* [47]. We also found that genes from the nicotinate and nicotinamide metabolism pathways were overrepresented among transcripts differentially expressed under high salt treatment in both leaves and roots.

Ethylene signalling also influences the response to salt stress in *Arabidopsis*. High salinity induces the accumulation and activation of EIN3/EIL1 protein. The activated EIN3 alleviates the accumulation of excess ROS accumulation and therefore increases salt tolerance of the plant [48]. We found that key enzymes in the cysteine and methionine, as well as glutathione, metabolism pathways were up-regulated under salt treatment in leaves but not roots. This suggests that ethylene and glutathione are preferentially synthesized in leaf tissue and play a role in long-term salt adaptation.

Several studies have demonstrated that transcription factors act as regulators of adaptation in plants. The functions of certain TFs (such as *AP2/EREBP*, *NAC*) in response to salt tolerance in particular have been well documented [35, 49–51]. We also observed that many TF encoding genes were up-regulated under increasing salt concentration. Transcription factors of *AP2*/*EREBP*, *WRKY*, *bZIP*, *NAC*, *MYB* and *bHLH* regulate downstream factors to involve in the major pathways, such as flavonoid biosynthesis, sulfur and glucosinolate metabolism [52–57]. This result confirms the GO enrichment and pathway enrichment analyses and shows an extensive regulation of transcription during salt adaptation. However, further investigations are required to find the exact regulatory networks of salt response in mangrove trees.

Natural selection is likely the major force driving *S. alba* salt adaptation to the fluctuating intertidal environment. We lacked comparative transcriptomic data to test how much of the gene expression response to salt treatment is the result of adaptive evolution. However, we did find a number of genes whose evolution at the amino acid level has either accelerated or shows signs of positive selection in the *S. alba* lineage (Additional file 8: Table S4). Indeed, *SA_12151*, a member of auxin-responsive family, responds to various abiotic stresses (auxin, high-salinity stresses) in rice [58]. *SA_26719* (peroxisomal 3-ketoacyl-CoA thiolase) is a peroxisome-associated gene. Previous studies have reported that salt stress affects peroxisome enzyme activities and protein expression [59]. Salt-induced expression of peroxisome-associated genes requires abscisic acid and other signaling pathways [60]. ABA is a stress hormone that plays a central role in a variety of abiotic stress responses. Finally, *SA_14625*, encoding a hydrolase, is involved in physiologically important processes in plants. It is up-regulated in response to high salinity stress in salt-tolerant rice [61]. Although our data suggest that these genes are involved in salt tolerance, the mechanisms driving evolution of these up-regulated PSGs must be further confirmed with physiological experiments. It would help us better understand the evolutionary dynamics of salt adaptation.

The most extreme challenge for species living in intertidal zones is the unstable and high salinity due to constant tidal fluctuations. Our analyses of DEGs point to the cellular environment as the possible site of molecular salt adaptation in *S. alba*. Thus, we suggest that the maintenance and regulation of cellular environmental homeostasis are important adaptive processes, contributing to evolutionary innovations.

## Conclusions

We performed comprehensive transcriptome analyses of *S. alba* leaves and roots across salinity conditions. The study demonstrates extensive transcriptional regulation of salt adaptation. Leaves and roots appear to adopt different dynamic gene regulation strategies for salt adaptation in *S. alba*. Most differentially expressed genes in leaves were well adapted to the medium saline condition. Our analyses report several candidate genes (including salt-related genes, TF-encoding genes, and PSGs) and major pathways including sulfur metabolism, flavone and flavonol biosynthesis, nicotinate and nicotinamide metabolism, and cysteine and methionine metabolism, that are involved in adaptation to high-salt environments. We deduce that the maintenance and regulation of cellular environmental homeostasis are important adaptive processes. Our study can also serve as a resource for future investigation of adaptive evolution in extreme environments. With additional investigations targeting the methylome and the proteome in the future, the exact mechanisms of salt adaptation in plants can be unveiled.

## Methods

### Plant materials and growth conditions

Eighteen *S. alba* seedlings (Additional file 9: Figure S5) were collected from the nursery of Dongzhai Harbor National Nature Reserve in Hainan with permission. All samples have been identified by Mr. Cairong Zhong, an officer of Dongzhai Harbor National Nature Reserve Administration. Then they were cultivated in the greenhouse (23 °C ~ 35 °C, 13.5-h day-length, a light intensity sufficient to allow normal growth) of Sun Yat-Sen University. The seedlings were transferred to sandy soil and grown using 1/2 Hoagland’s solution for 3 weeks to help plants adapt to the new environment. The seedlings were divided into three groups, each group containing two biological replicates. These three experimental groups were irrigated using solutions containing 0, 250, or 500 mM NaCl for 7 days. In natural condition, *S. alba* is usually found near the estuary. The rainwater and upstream rivers give it little exposure to normal seawater concentrations. *S. alba* reaches an optimal growth in 5 to 50% seawater. Medium saline (250 mM NaCl), approximately half of the normal seawater salinity, is close to the upper bound for natural *S. alba* growth [19]. Although 500 mM and 0 mM provide the hyper-saline (stressful) and low-saline conditions, respectively, 0–250 mM is a range well tolerated by mangroves in nature. In contrast, 250–500 mM is genuinely stressful. The treatment duration was based on our previous study [62].

### Sample collection, sequencing, and alignment

Healthy young leaves and roots of each seedling were sampled after 7 days and immediately frozen in liquid nitrogen and stored at − 80 °C. Three plants from the same group were pooled to minimize variation among individual plants. Total RNA was extracted using the Plant RNA kit (Omega Bio-Tek, Doraville, USA) according to the manufacturer’s instructions. For each sample, at least 20 μg of total RNA was sent to the Novogene Bioinformatics Technology Co., Ltd. for sequencing using the Illumina HiSeq 2000 platform. Raw sequence reads were first purified by trimming adapter sequences. In order to reduce sequencing error, paired-end low-quality reads were removed by customized scripts if they met the following criteria: i) they comprised more than 5% unknown (N) bases; ii) comprised more than 20% bases with quality <= 10; iii) average quality was under 20. The last base of the filtered reads was also trimmed according to the results of FastQC.

Clean reads from each sample were aligned to the *S. alba* reference genome [10] using TopHat v2.1.1 [63] and Bowtie2 v2.2.9 [64]. We then performed the genome-guided transcriptome assembly and differential transcript expression analyses using Cufflinks v2.2.1 [65]. We reported expression levels as Fragments Per Kilobase of exon model per Million mapped reads (FPKM) values. We then calculated the Pearson’s correlation coefficients between all replicates using gene expression data.

### Identification of differentially expressed genes and expression pattern analyses

We identified DEGs from two comparisons: 250 mM NaCl vs 0 mM NaCl and 500 mM NaCl vs 250 mM NaCl. We considered transcripts with *q*-values (FDR-adjusted *P*-values) below 5% and fold change higher than 2 in either comparison as differentially expressed. We called genes whose expression increased with salt concentration “up-regulated” and transcripts with change in the opposite direction “down-regulated.” The remaining genes were denoted as “no significant difference”. These DEGs were grouped into eight distinct and representative clusters (LC1 ~ LC8, RC1 ~ RC8) via pairwise comparisons between three expression patterns (up-regulated, down-regulated, or non-significant difference) in leaves and roots. To determine which clusters have statistically significant enrichments in the number of genes assigned, we used a permutation test with 10,000 runs. In each permutation we first shuffled all expression quantities obtained under different conditions randomly to destroy any association between the conditions and expression quantities and assigned them to their original gene groups. We then carried out the cluster analysis as for the real data and recorded whether the number of genes assigned to every cluster in the permutation was no smaller than in the actual observation. After 10,000 permutation runs, we estimated the frequency of assigning no fewer genes to a cluster than what is seen in the real expression data, allowing for significance testing.

### Gene annotation and ontology enrichment analyses

We obtained *S. alba* genome annotations and assigned gene ontology (GO) and KEGG orthology (KO) terms to all genes. GO terms were assigned using the Web Gene Ontology Annotation Plotting (WEGO 2.0) [66] and visualized by Cytoscape_v3.7.2 [67]. Transcription factor annotations for *S. alba* and its relatives with genome sequences: *Sonneratia caseolaris* (true mangrove) [10], *Punica granatum* (non-mangrove) [68], and *Eucalyptus grandis* (non-mangrove) [69] were performed using iTAK1.7 [70]. We tested for enrichment of TFs among differentially expressed *S. alba* genes using Fisher’s exact test. KEGG annotation was carried out to assign DEGs to metabolic pathways. We assigned all up-regulated genes in leaves and roots to KEGG pathways and compared these pathways’ gene numbers with the whole genome background. KEGG pathway data were from the R package clusterProfiler [71] which supports downloading the latest KEGG annotations. Pathways with more than three up-regulated genes were processed using Fisher’s exact test. Enrichment was declared significant if Benjamini-Hochberg corrected *P-*values fell below 5%. We identified key salt-responsive genes from *S. alba* genome annotations and BLASTP searches of genes up-regulated in *S. alba* against *Arabidopsis thaliana* genome data (TAIR10, www.arabidopsis.org) with cut-off *e*-value < 10^− 6^.

### Selection analyses

In order to detect up-regulated genes that might undergo positive selection in *S. alba*, we first identified high-quality orthologs within *S. alba* and its relatives, including *Punica granatum*, *Trapa bispinosa*, *Lagerstroemia speciosa*, *Duabanga grandiflora*, and *Eucalyptus grandis*. These species belong to the same order Myrtales (APG IV, www.mobot.org/MOBOT/research/APweb/welcome.html). The whole genome sequences of *Punica granatum*, and *Eucalyptus grandis* were downloaded from phytozome and NCBI [68, 69]. For the more closely related species *Trapa bispinosa*, *Lagerstroemia speciosa*, and *Duabanga grandiflora*, the transcriptomes were sequenced in our previous study [72]. Using the orthologs identified by OrthoMCL [73] as an initial data set, we used the reciprocal BLASTP best-hit method for *S. alba* and five related species. We then individually aligned orthologous proteins using MUSCLE [74] and used the aligned protein sequences to generate codon alignments using PAL2NAL [75]. Alignments shorter than 150 bp after removing sites with ambiguous data were discarded. This process yielded 3936 high-quality orthologs within *S. alba* and its relatives. These codon alignments together with a phylogenetic tree [76] were used for subsequent analyses.

We calculated Ka/Ks ratio for each branch of the phylogenetic tree using the “free-ratio” model in the PAML package [77]. All orthologs were concatenated into a single super gene. We used the branch model in the PAML package to identify positively selected genes, setting the *S. alba* branch as the foreground [78]. We introduced the likelihood comparison between the alternative model allowing the foreground branch to evolve under a different rate and the null model assuming that all branches have been evolving at the same rate for each ortholog set. We also used the branch-site model in PAML by setting model = 2, NSsites = 2 [79]. The comparison of likelihoods between the alternative model assuming that at least one site in the foreground has undergone positive selection and the null model that assumes none was used as the test of positive selection. The likelihood ratio test method was the same as for the branch model. Multiple testing was corrected by applying the Benjamini-Hochberg method implemented in the R function *p.adjust*. We considered the up-regulated genes with FDR below 5% as PSGs. Finally, we manually filtered all PSGs with potential errors in their alignments to minimize the false positive rate.

### RNA-seq data validation with real-time quantitative PCR

The qRT-PCR was performed to validate the RNA-seq results for 16 transcripts from 16 distinct clusters (*SA_28450* both from LC8 and RC5) in leaves and roots. Gene-specific primers for qRT-PCR were designed using the Primer Premier software and are listed in Additional file 10: Table S5.

First-strand cDNAs were synthesized from 1 μg of total RNA using the PrimeScript™ RT reagent Kit with gDNA Eraser (TaKaRa). The expression of the β-actin gene served as the internal control. qRT-PCR was carried out using the Real-Time PCR system on an ABI 7900HT with the following program: 95 °C for 30 s (95 °C for 5 s, 55 °C for 10 s and 72 °C for 15 s), 40 cycles. Each sample was analysed with three technical replicates. The relative expression profiles of selected genes were calculated using the 2^-△△CT^ method [80]. Correlations between expression profiles of selected genes from RNA-seq and qRT-PCR were estimated using a simple linear regression model using the *lm* function in R.

## Supplementary information

**Additional file 1: Figure S1.** Gene expression correlations among all samples. Pearson’s correlation plot visualizes the correlation coefficients. Scale bar represents the range of the value displayed. The correlation coefficients between biological replicates in Leaf 0 mM, Leaf 250 mM, Leaf 500 mM, Root 0 mM, Root 250 mM, and Root 500 mM conditions are 0.92, 0.97, 0.90, 0.97, 0.87, 0.90, respectively.

**Additional file 2: Figure S2.** Cluster analysis and permutation test of differentially expressed genes (DEGs). Expression profiles of DEGs under different salinity contrasts are shown for eight clusters in leaves and roots. DEGs in leaves are significantly enriched in three clusters (LC8, LC4, LC5), whereas DEGs in roots are significantly enriched in RC1 and RC5. In each cluster, the black line represents the expression level (means of log_10_(FPKM value + 1)) of all enriched genes, the error bars represent standard deviations. A permutation test was used to evaluate the significance of differences. The clusters with statistically significant difference in the number of DEGs are marked with triple (*P*-value < 0.01) asterisks. The observed and expected (only significant clusters) numbers of genes belonging to each cluster are labeled on the top of each cluster.

**Additional file 3: Table S1.** The GO annotation of DEGs. URG represents up-regulated gene; DRG represents down-regulated gene. The numbers in the brackets are percentages of genes with GO ID and those outside the brackets are gene numbers.

**Additional file 4: Table S2.** Summary of GO terms enriched among genes in co-expression clusters.

**Additional file 5: Table S3.** The KEGG annotation of up-regulated genes. URG represents up-regulated gene. The numbers in the brackets are percentages of genes with KO ID and those outside the brackets are gene or pathway numbers.

**Additional file 6: Figure S3.** Different expression patterns of transcription factor genes in leaves (A) and roots (B) across salinity contrasts (0, 250 and 500 mM NaCl). Various TF families showing differential expression patterns under different conditions are given on the right side of heat map. The middle-upper scale represents FPKM values with z-score normalization.

**Additional file 7: Figure S4.** Ka/Ks ratio along each branch of the phylogenetic tree. The mangrove lineage (*S. alba*) is colored red, set as the foreground.

**Additional file 8: Table S4.** PSGs Related to Salt Adaptation in *Sonneratia alba*.

**Additional file 9: Figure S5.** The seedlings of *S. alba*.

**Additional file 10: Table S5.** Primer sequences for real-time quantitative PCR analysis.

## Data Availability

The RNA-seq data have been deposited in the Genome Sequence Archive database (Accession No. PRJCA001412) and the NCBI Sequence Read Archive database (Accession No. PRJNA615770).

## Abbreviations

AOC: Allene oxide cyclase
DEGs: Differentially expressed genes
FPKM: Fragments Per Kilobase of exon model per Million mapped reads
GO: Gene Ontology
KO: KEGG orthology
NHX: Sodium/hydrogen exchanger
PIP: Plasma membrane intrinsic protein
PSGs: Positively selected genes
qPCR: Real-time quantitative PCR
ROS: Reactive oxygen species
SOS: Salt overly sensitive
TFs: Transcription factors
TIP: Tonoplast intrinsic protein
UV: Ultraviolet
WEGO: Web Gene Ontology Annotation Plotting
