# Supplementary material for: Molecular adaptation to salinity fluctuation in tropical intertidal environments of a mangrove tree Sonneratia alba
Source: BMC Plant Biol. 2020 Apr 22;20:178. doi: 10.1186/s12870-020-02395-3 (PMC7178616; doi:10.1186/s12870-020-02395-3)
Supplement: Supplementary file 1 — Additional file 1: Figure S1. Gene expression correlations among all samples. Pearson’s correlation plot visualizes the correlation coefficients. Scale bar represents the range of the value displayed. The correlation coefficients between biological replicates in Leaf 0 mM, Leaf 250 mM, Leaf 500 mM, Root 0 mM, Root 250 mM, and Root 500 mM conditions are 0.92, 0.97, 0.90, 0.97, 0.87, 0.90, respectively. [file 12870_2020_2395_MOESM1_ESM.docx]

**Additional file 1: Figure S1.** Gene expression correlations among all samples. Pearson’s correlation plot visualizes the correlation coefficients. Scale bar represents the range of the value displayed. The correlation coefficients between biological replicates in Leaf 0 mM, Leaf 250 mM, Leaf 500 mM, Root 0 mM, Root 250 mM, and Root 500 mM conditions are 0.92, 0.97, 0.90, 0.97, 0.87, 0.90, respectively.
