# Supplementary material for: Molecular adaptation to salinity fluctuation in tropical intertidal environments of a mangrove tree Sonneratia alba
Source: BMC Plant Biol. 2020 Apr 22;20:178. doi: 10.1186/s12870-020-02395-3 (PMC7178616; doi:10.1186/s12870-020-02395-3)
Supplement: Supplementary file 2 — Additional file 2: Figure S2. Cluster analysis and permutation test of differentially expressed genes (DEGs). Expression profiles of DEGs under different salinity contrasts are shown for eight clusters in leaves and roots. DEGs in leaves are significantly enriched in three clusters (LC8, LC4, LC5), whereas DEGs in roots are significantly enriched in RC1 and RC5. In each cluster, the black line represents the expression level (means of log10(FPKM value + 1)) of all enriched genes, the error bars represent standard deviations. A permutation test was used to evaluate the significance of differences. The clusters with statistically significant difference in the number of DEGs are marked with triple (P-value < 0.01) asterisks. The observed and expected (only significant clusters) numbers of genes belonging to each cluster are labeled on the top of each cluster. [file 12870_2020_2395_MOESM2_ESM.docx]

**Additional file 2: Figure S2.** Cluster analysis and permutation test of differentially expressed genes (DEGs). Expression profiles of DEGs under different salinity contrasts are shown for eight clusters in leaves and roots. DEGs in leaves are significantly enriched in three clusters (LC8, LC4, LC5), whereas DEGs in roots are significantly enriched in RC1 and RC5. In each cluster, the black line represents the expression level (means of log_10_(FPKM value +1)) of all enriched genes, the error bars represent standard deviations. A permutation test was used to evaluate the significance of differences. The clusters with statistically significant difference in the number of DEGs are marked with triple (*P*-value < 0.01) asterisks. The observed and expected (only significant clusters) numbers of genes belonging to each cluster are labeled on the top of each cluster.
