# Supplementary material for: Molecular adaptation to salinity fluctuation in tropical intertidal environments of a mangrove tree Sonneratia alba
Source: BMC Plant Biol. 2020 Apr 22;20:178. doi: 10.1186/s12870-020-02395-3 (PMC7178616; doi:10.1186/s12870-020-02395-3)
Supplement: Supplementary file 3 — Additional file 3: Table S1. The GO annotation of DEGs. URG represents up-regulated gene; DRG represents down-regulated gene. The numbers in the brackets are percentages of genes with GO ID and those outside the brackets are gene numbers. [file 12870_2020_2395_MOESM3_ESM.docx]

**Additional file 3: Table S1.** The GO annotation of DEGs.

| **Condition** | **URG with GO ID** | **DRG with GO ID** |
| --- | --- | --- |
| Leaf  (0mM vs 250mM) | 181 (46.77%) | 382 (52.62%) |
| Root  (0mM vs 250mM) | 95 (56.21%) | 191 (64.75%) |
| Leaf  (250mM vs 500mM) | 563 (53.26%) | 728 (56.17%) |
| Root  (250mM vs 500mM) | 139 (56.28%) | 70 (52.63%) |

URG represents up-regulated gene; DRG represents down-regulated gene. The numbers in the brackets are percentages of genes with GO ID and those outside the brackets are gene numbers.
