# Supplementary material for: Molecular adaptation to salinity fluctuation in tropical intertidal environments of a mangrove tree Sonneratia alba
Source: BMC Plant Biol. 2020 Apr 22;20:178. doi: 10.1186/s12870-020-02395-3 (PMC7178616; doi:10.1186/s12870-020-02395-3)
Supplement: Supplementary file 4 — Additional file 4: Table S2. Summary of GO terms enriched among genes in co-expression clusters. [file 12870_2020_2395_MOESM4_ESM.docx]

**Additional file 4: Table S2.** Summary of GO terms enriched among genes in co-expression clusters.

| **Cluster (Gene Number)** | **Enriched GO BP, CC, MF terms** | **No. of genes in the cluster (%)** | **No. of genes in the background (%)** | **FDR** |
| --- | --- | --- | --- | --- |
| LC4-RC4 (67) | **response to stress ^BP^** | 12 (38.7) | 1155 (11.3) | 2.82E-02 |
|  | **acetyltransferase complex ^CC^** | 1 (6.7) | 25 (0.3) | 4.76E-02 |
|  | **ion binding ^MF^** | 17 (56.7) | 3649 (32.0) | 4.75E-02 |
|  | └cation binding | 11 (36.7) | 1943 (17.0) | 4.75E-02 |
|  | └metal ion binding | 11 (36.7) | 1930 (16.9) | 4.75E-02 |
|  | **transmembrane transporter activity ^MF^** | 6 (20.0) | 682 (6.0) | 4.75E-02 |
|  | **oxidoreductase activity ^MF^** | 14 (46.7) | 1697 (14.9) | 4.69E-03 |
|  | └electron transfer activity | 4 (13.3) | 236 (2.1) | 4.75E-02 |
|  | └oxidoreductase activity, acting on paired donors, with  incorporation or reduction of molecular oxygen | 4 (13.3) | 272 (2.4) | 4.75E-02 |
|  | └oxidoreductase activity, acting on the CH-NH2 group of  donors | 2 (6.7) | 32 (0.3) | 4.75E-02 |
|  | └oxidoreductase activity, acting on the CH-CH group of  donors | 4 (13.3) | 226 (2.0) | 4.75E-02 |
|  | └oxidoreductase activity, acting on the CH-CH group of  donors, NAD or NADP as acceptor | 4 (13.3) | 182 (1.6) | 4.75E-02 |
|  | └2-alkenal reductase [NAD(P)+] activity | 3 (10.0) | 146 (1.3) | 4.75E-02 |
|  | **N-acyltransferase activity ^MF^** | 2 (6.7) | 47 (0.4) | 4.75E-02 |
|  | └N-acetyltransferase activity | 2 (6.7) | 45 (0.4) | 4.75E-02 |
| LC4 (965) | **response to stimulus ^BP^** | 141 (36.3) | 2568 (25.0) | 1.57E-04 |
|  | └response to stress | 79 (20.4) | 1155 (11.3) | 9.56E-05 |
|  | └respiratory burst | 6 (1.5) | 10 (0.1) | 1.57E-04 |
|  | └respiratory burst involved in defense response | 6 (1.5) | 10 (0.1) | 1.57E-04 |
|  | └response to biotic stimulus | 31 (8.0) | 337 (3.3) | 6.12E-04 |
|  | └response to chemical | 75 (19.3) | 1139 (11.1) | 2.01E-04 |
|  | └response to inorganic substance | 38 (9.8) | 452 (4.4) | 5.01E-04 |
|  | └response to drug | 24 (6.2) | 212 (2.1) | 2.97E-04 |
|  | **oxidoreductase activity ^MF^** | 99 (23.5) | 1697 (14.86) | 6.00E-04 |
| LC8 (1045) | **carbohydrate metabolic process ^BP^** | 102 (22.1) | 1038 (10.1) | 4.67E-12 |
|  | └oligosaccharide metabolic process | 48 (10.4) | 341 (3.3) | 1.67E-10 |
|  | └disaccharide metabolic process | 48 (10.4) | 333 (3.2) | 7.69E-11 |
|  | └sucrose metabolic process | 46 (10.0) | 304 (3.0) | 5.46E-11 |
|  | └cellular carbohydrate metabolic process | 63 (13.6) | 538 (5.2) | 1.83E-10 |
|  | └polysaccharide metabolic process | 62 (13.4) | 466 (4.5) | 4.67E-12 |
|  | └glucan metabolic process | 56 (12.1) | 399 (3.9) | 4.67E-12 |
|  | └cellular glucan metabolic process | 56 (12.1) | 399 (3.9) | 4.67E-12 |
|  | └starch metabolic process | 48 (10.4) | 329 (3.2) | 5.55E-11 |
|  | └cellular polysaccharide metabolic process | 60 (13.0) | 451 (4.4) | 4.67E-12 |
|  | **negative regulation of molecular function ^BP^** | 15 (3.2) | 82 (0.8) | 2.87E-04 |
|  | └negative regulation of catalytic activity | 15 (3.2) | 79 (0.8) | 1.90E-04 |
|  | **nucleotide-sugar metabolic process ^BP^** | 13 (2.8) | 59 (0.6) | 1.72E-04 |
|  | └UDP-glucose metabolic process | 10 (2.2) | 32 (0.3) | 9.63E-05 |
|  | **membrane ^CC^** | 186 (57.1) | 3512 (45.5) | 4.11E-04 |
|  | **extracellular region ^CC^** | 42 (12.9) | 425 (5.5) | 7.77E-06 |
|  | **cell periphery ^CC^** | 108 (33.1) | 1647 (21.4) | 1.17E-05 |
|  | └external encapsulating structure | 51 (15.6) | 392 (5.1) | 2.22E-11 |
|  | └cell wall | 51 (15.6) | 390 (5.1) | 2.22E-11 |
|  | └plant-type cell wall | 25 (7.7) | 145 (1.9) | 8.45E-08 |
|  | **catalytic activity ^MF^** | 406 (79.1) | 7821 (68.5) | 4.90E-06 |
|  | └hydrolase activity, acting on glycosyl bonds | 43 (8.4) | 379 (3.3) | 4.90E-06 |
|  | └hydrolase activity, hydrolyzing O-glycosyl compounds | 39 (7.6) | 330 (2.9) | 4.90E-06 |
|  | └glucosyltransferase activity | 24 (4.7) | 151 (1.3) | 7.80E-06 |
|  | └cellulose synthase activity | 10 (1.9) | 31 (0.3) | 5.04E-05 |
|  | └cellulose synthase (UDP-forming) activity | 10 (1.9) | 31 (0.3) | 5.04E-05 |

*LC4-RC4: Overlaps between LC4 and RC4; LC8-RC8: Overlaps between LC8 and RC8.

LC8-RC8: No significant enrichment of GO terms was found.

BP: Biological Process; CC: Cellular Component; MF: Molecular Function.
