# Supplementary material for: Molecular adaptation to salinity fluctuation in tropical intertidal environments of a mangrove tree Sonneratia alba
Source: BMC Plant Biol. 2020 Apr 22;20:178. doi: 10.1186/s12870-020-02395-3 (PMC7178616; doi:10.1186/s12870-020-02395-3)
Supplement: Supplementary file 5 — Additional file 5: Table S3. The KEGG annotation of up-regulated genes. URG represents up-regulated gene. The numbers in the brackets are percentages of genes with KO ID and those outside the brackets are gene or pathway numbers. [file 12870_2020_2395_MOESM5_ESM.docx]

**Additional file 5: Table S3.** The KEGG annotation of up-regulated genes.

| **Condition** | **URG with KO ID** | **No. of enriched KEGG pathway** |
| --- | --- | --- |
| Leaf  (0mM vs 250mM) | 308 (79.59%) | 98 |
| Root  (0mM vs 250mM) | 153 (90.53%) | 52 |
| Leaf  (250mM vs 500mM) | 920 (87.04%) | 222 |
| Root  (250mM vs 500mM) | 207 (83.81%) | 126 |

URG represents up-regulated gene. The numbers in the brackets are percentages of genes with KO ID and those outside the brackets are gene or pathway numbers.
