# Supplementary material for: Molecular adaptation to salinity fluctuation in tropical intertidal environments of a mangrove tree Sonneratia alba
Source: BMC Plant Biol. 2020 Apr 22;20:178. doi: 10.1186/s12870-020-02395-3 (PMC7178616; doi:10.1186/s12870-020-02395-3)
Supplement: Supplementary file 6 — Additional file 6: Figure S3. Different expression patterns of transcription factor genes in leaves (A) and roots (B) across salinity contrasts (0, 250 and 500 mM NaCl). Various TF families showing differential expression patterns under different conditions are given on the right side of heat map. The middle-upper scale represents FPKM values with z-score normalization. [file 12870_2020_2395_MOESM6_ESM.docx]

**Additional file 6: Figure S3.** Different expression patterns of transcription factor genes in leaves (A) and roots (B) across salinity contrasts (0, 250 and 500 mM NaCl). Various TF families showing differential expression patterns under different conditions are given on the right side of heat map. The middle-upper scale represents FPKM values with z-score normalization.
