# Supplementary material for: Molecular adaptation to salinity fluctuation in tropical intertidal environments of a mangrove tree Sonneratia alba
Source: BMC Plant Biol. 2020 Apr 22;20:178. doi: 10.1186/s12870-020-02395-3 (PMC7178616; doi:10.1186/s12870-020-02395-3)
Supplement: Supplementary file 7 — Additional file 7: Figure S4. Ka/Ks ratio along each branch of the phylogenetic tree. The mangrove lineage (S. alba) is colored red, set as the foreground. [file 12870_2020_2395_MOESM7_ESM.docx]

**Additional file 7: Figure S4.** Ka/Ks ratio along each branch of the phylogenetic tree. The mangrove lineage (*S. alba*) is colored red, set as the foreground.
