# Supplementary material for: Molecular adaptation to salinity fluctuation in tropical intertidal environments of a mangrove tree Sonneratia alba
Source: BMC Plant Biol. 2020 Apr 22;20:178. doi: 10.1186/s12870-020-02395-3 (PMC7178616; doi:10.1186/s12870-020-02395-3)
Supplement: Supplementary file 8 — Additional file 8: Table S4. PSGs Related to Salt Adaptation in Sonneratia alba. [file 12870_2020_2395_MOESM8_ESM.docx]

**Additional file 8: Table S4.** PSGs Related to Salt Adaptation in *Sonneratia alba*.

| **Gene ID** | **Condition** | ***A. thaliana*** | **Annotation** | ***P* value** | **FDR** |
| --- | --- | --- | --- | --- | --- |
| **Using Branch Model** | | | | | |
| *SA_00884* | Leaf  (0mM vs 250mM) | *AT5G51970* | sorbitol dehydrogenase | 2.63E-03 | 4.50E-02 |
| *SA_02803* | Leaf  (0mM vs 250mM) | *AT4G34480* | glucan endo-1,3-beta-glucosidase | 2.70E-03 | 4.58E-02 |
| *SA_26719* | Leaf  (250mM vs 500mM) | *AT2G33150* | peroxisomal 3-ketoacyl-CoA thiolase | 6.88E-07 | 1.18E-04 |
| *SA_04140* | Leaf  (250mM vs 500mM) | *AT1G59650* | CW14 protein | 4.82E-06 | 5.13E-04 |
| *SA_13519* | Leaf  (250mM vs 500mM) | *AT2G23450* | protein kinase superfamily protein | 2.10E-05 | 1.59E-03 |
| *SA_27383* | Leaf  (250mM vs 500mM) | *AT3G20300* | extracellular ligand-gated ion channel protein | 3.29E-05 | 2.02E-03 |
| *SA_12184* | Leaf  (250mM vs 500mM) | *AT4G14040* | selenium-binding protein | 5.39E-04 | 1.63E-02 |
| *SA_22704* | Leaf  (250mM vs 500mM) | *AT1G62300* | WRKY family transcription factor | 5.65E-04 | 1.65E-02 |
| *SA_17616* | Leaf  (250mM vs 500mM) | *AT1G05300* | zinc transporter 5 precursor | 6.74E-04 | 1.78E-02 |
| *SA_12151* | Leaf  (250mM vs 500mM) | *AT5G47530* | auxin-responsive family protein | 9.14E-04 | 2.20E-02 |
| *SA_01381* | Leaf  (250mM vs 500mM) | *AT4G21490* | NAD(P)H dehydrogenase B3 | 1.80E-03 | 3.61E-02 |
| *SA_08107* | Leaf  (250mM vs 500mM) | *AT3G03440* | ARM repeat superfamily protein | 2.20E-03 | 4.09E-02 |
| *SA_29168* | Leaf  (250mM vs 500mM) | *AT2G27500* | glucan endo-1,3-beta-glucosidase | 2.47E-03 | 4.33E-02 |
| *SA_07962* | Leaf  (250mM vs 500mM) | *AT5G62350* | plant invertase/pectin methylesterase inhibitor superfamily protein | 2.86E-03 | 4.79E-02 |
| **Using Branch-site Model** | | | | | |
| *SA_14625* | Leaf  (0mM vs 250mM) | *AT5G15870* | glycosyl hydrolase family 81 protein | 1.62E-05 | 5.85E-04 |
| *SA_26719* | Leaf  (250mM vs 500mM) | *AT2G33150* | peroxisomal 3-ketoacyl-CoA thiolase | 1.11E-16 | 2.57E-14 |
