# Supplementary figures and images for: Molecular adaptation to salinity fluctuation in tropical intertidal environments of a mangrove tree Sonneratia alba
Source: BMC Plant Biol. 2020 Apr 22;20:178. doi: 10.1186/s12870-020-02395-3 (PMC7178616; doi:10.1186/s12870-020-02395-3)

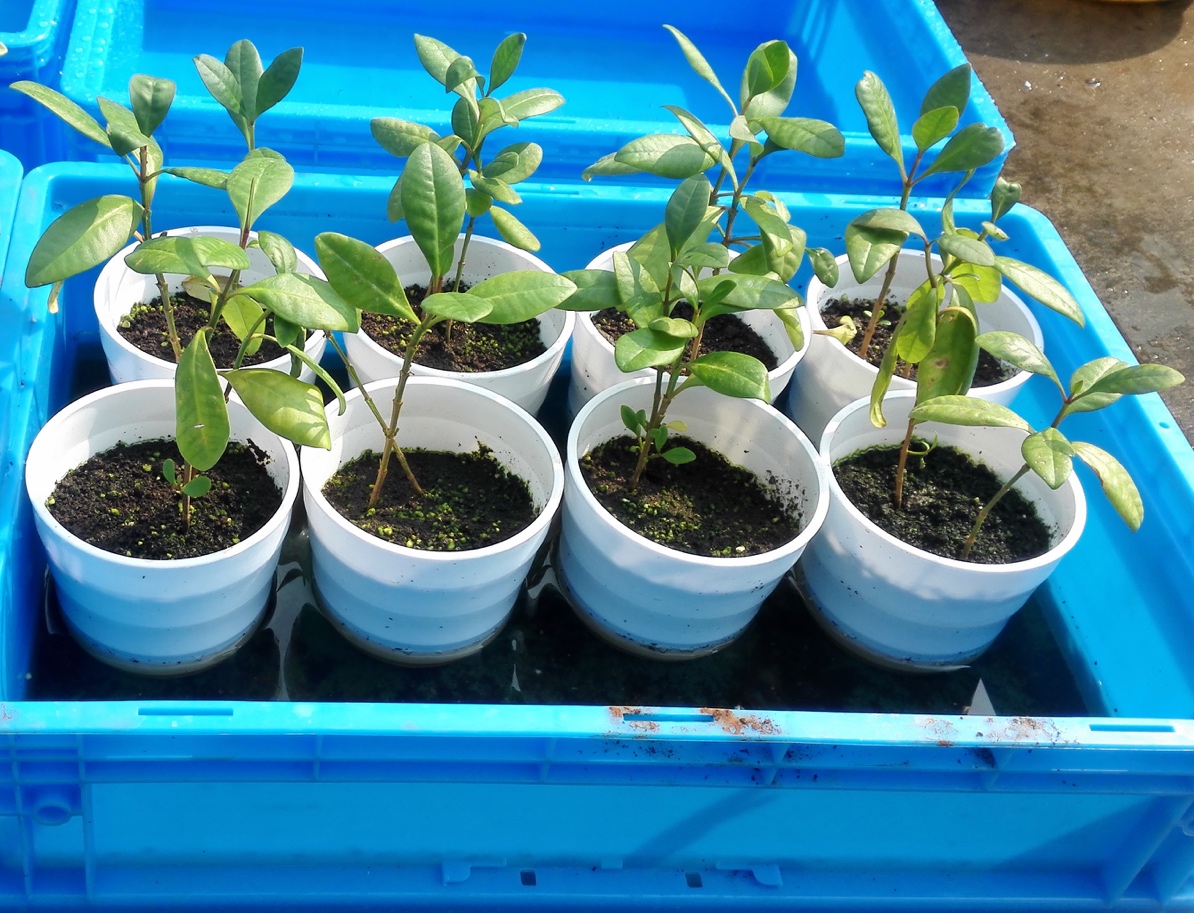


**Additional file 9: Figure S5.** The seedlings of *S. alba*.

Supplement: Supplementary file 9 — Additional file 9: Figure S5. The seedlings of S. alba. [file 12870_2020_2395_MOESM9_ESM.docx]
