# Supplementary material for: Molecular adaptation to salinity fluctuation in tropical intertidal environments of a mangrove tree Sonneratia alba
Source: BMC Plant Biol. 2020 Apr 22;20:178. doi: 10.1186/s12870-020-02395-3 (PMC7178616; doi:10.1186/s12870-020-02395-3)
Supplement: Supplementary file 10 — Additional file 10: Table S5. Primer sequences for real-time quantitative PCR analysis. [file 12870_2020_2395_MOESM10_ESM.docx]

**Additional file 10: Table S5.** Primer sequences for real-time quantitative PCR analysis.

| **Gene** | **Sense primer (5’-3’)** | **Antisense primer (5’-3’)** |
| --- | --- | --- |
| *SA_23236* | GAACCTCTTCGTGGCACTTC | TCATCCCAGTGGTCAGCAAA |
| *SA_20230* | GTCTCTCTCACCAGCTCCAG | GGCGATGGAATTTGTTGGGA |
| *SA_06679* | CCCACCATCTCCTCCAACAA | CGGTTTCCCCTTCAATGGTG |
| *SA_14631* | CCAGCTCAACCTTTCATCGG | GGTCCCCGCTGTAAACTTTG |
| *SA_24172* | AAGCCGGCTAAGGATCTCTC | TGCAAGAATCCGGAACTCCT |
| *SA_07877* | CCCGGCTTTTGTCTTTGGAT | CCTCATCCTGATCTCGTGCT |
| *SA_16609* | TACCCAACCAAGAAGTCGCT | TGAGCTTGCGGTTGTTCATC |
| *SA_28450* | CGTGAAGAAAGTGTACGGGG | TCGTACTTGTCCAGCTCGTT |
| *SA_03021* | ACCTCTACAAGAAGATCGCCA | GGTCGCGGAAACTGAAGAAG |
| *SA_17026* | TGCTCCATTTGTCTCCCTCT | CCTGACCAACCATTCAAGACG |
| *SA_16777* | GCCACGTATTTGCTGCCTTA | TTCAAGTGTGCCCGCTCATA |
| *SA_07445* | ATCTTCGTGACCAGGTCCAG | TCTGCAAATCCCCAATTCGC |
| *SA_18291* | TCCGATGCCTCACTTCTGTT | TTGACTGAGGCCTTGTCCAA |
| *SA_10206* | GTCTGGGGCTGGGAATAACT | ACCTTAGGAGACGGGAACAC |
| *SA_20653* | GTTCCAGGTCCAAGGCAATG | TTTGTCTGCTGGCCCTTTTC |
